# Current Strategies for Limb Salvage and Reconstruction in Pediatric Lower Extremity Malignant Bone Tumors: Focus on Growth Preservation and Functional Outcomes

**DOI:** 10.3390/children12121700

**Published:** 2025-12-16

**Authors:** Zhu Liu, Haoqi Cai, Yuchan Li, Zhigang Wang

**Affiliations:** Department of Orthopedic Surgery, Shanghai Children’s Medical Center, School of Medicine, Shanghai Jiao Tong University, Shanghai 200127, China; liuzhu@scmc.com.cn (Z.L.);

**Keywords:** pediatric bone sarcoma, limb salvage, rotationplasty, growth-plate preservation, 3D-printed endoprosthesis

## Abstract

**Highlights:**

**What are the main findings?**

- Rotationplasty is particularly suitable for children under 6 years with periarticular tumors, offering stable reconstruction, low complication rates, and functional benefits, though cosmetic concerns may arise in adolescence.
- Endoprosthetic reconstruction, especially extendable and 3D-printed options, is effective for larger defects in children aged 6–12, preserving growth potential and achieving high prosthesis survival rates (e.g., 87.4% at 5 years for proximal tibial cases), but often requires multiple revisions.

**What are the implications of the main findings?**

- Personalized reconstruction strategies that account for age, defect size, and growth- plate preservation can optimize tumor-free survival, limb function, and quality of life, shifting away from amputation toward limb salvage.
- Emerging technologies like 3D-printed joint-preserving prostheses may reduce complications such as nonunion and infection, enabling better long-term outcomes in resource-limited settings and skeletally immature patients.

**Abstract:**

**Background/Objectives:** Osteosarcoma and Ewing sarcoma are the predominant malignant bone tumors of the lower limbs in children. With 5-year survival rates of 70–77% for localized disease, limb salvage with growth-compatible reconstruction has replaced amputation as the standard. This review aimed to synthesize current reconstruction strategies, propose an age-and defect-based decision algorithm, and highlight growth-preserving innovations for skeletally immature patients. **Methods:** This narrative review of surgical techniques—including rotationplasty, biological reconstruction (vascularized/non-vascularized fibula, allograft, recycled autograft, “hot dog” composite), bone transport, and endoprosthetic replacement (modular, extendable, 3D-printed)—was conducted, with a literature search covering January 1990 to October 2025 and emphasized pediatric studies published after 2020, emphasizing pediatric outcomes, complication profiles, and functional scores. **Results:** Across pediatric and mixed-age cohorts (typically *n* ≈ 10–30 per technique; median follow-up 3–10 years), rotationplasty demonstrated high durability with Musculoskeletal Tumor Society (MSTS) scores of 21–28/30, especially in children < 6 years. Biological reconstruction achieved >80% union in defects < 6 cm, while vascularized fibula grafts yielded 82–95% union for 6–15 cm defects. Bone transport produced reliable union for 3–15 cm defects but required prolonged fixation (40–60 days/cm) and had high pin-tract infection rates (50–60%). Extendable endoprostheses demonstrated 5-year prosthesis survival of 54–87%, while early joint-preserving 3D-printed implants improved MSTS scores from 17 to 28 points in a pediatric series (*n* = 7, mean follow-up 30 months). **Conclusions:** Personalized reconstruction guided by a child-centered algorithm optimizes oncologic control, skeletal growth, and long-term function. Emerging 3D-printed joint-preserving implants and noninvasive lengthening technologies promise further reduction in revisions and complications in pediatric limb salvage.

## 1. Introduction

The incidence of primary malignant bone tumors in children is approximately 4.4 per million, accounting for 5% of all childhood cancers [1]. Ewing sarcoma (EWS) and osteosarcoma (OSC) are the most common primary malignant bone tumors in children and adolescents [2,3]. The annual incidence in children under 10 years of age is 1.7 per 100,000, whereas the annual incidence in patients aged 10 to 19 years increases to 8.2 per 100,000 [4]. The two main sites of Ewing sarcoma are the long bones of the lower extremities (29%) and the axial bones (45%). Among osteosarcomas, the most common site is the long bones of the lower extremity (78%), with the axial region accounting for less than 10% of cases [5]. Over the past few decades, great progress has been made in the treatment of childhood and adolescent cancer. According to the latest SEER data (2015–2021), the 5-year relative survival rate of patients with localized osteosarcoma lesions is 77%, the 5-year relative survival rate of patients with regional lesions (lesions beyond the bone but without distant metastasis) is 60%, and the 5-year relative survival rate of patients with lesions with distant metastasis at diagnosis is only 25%. The overall 5-year survival rate of all stages is 59% [6,7]. In contemporary EURO-EWING99/EWING 2008 protocols, patients with localized high-risk Ewing sarcoma achieve approximately 60% 3-year event-free survival (EFS) and 74% 3-year overall survival (OS), with BuMel consolidation improving 3-year EFS to ~67% and OS to ~78%; in contrast, patients with isolated pulmonary or pleural metastases reach 3-year EFS of about 51–57% and 3-year OS around 68%, and long-term survival remains unsatisfactory [8,9,10,11]. According to the phase III Ewing 2008R1 Trial, 3-year EFS for standard-risk localized Ewing sarcoma reached 81.7% and 3-year OS 94.6% [12]. The lower limb function and quality of life of these patients depend to a certain extent on the choice of surgery. Before effective adjuvant therapy, amputation is the main means of treatment, but amputation has a certain impact on quality of life and function [13]. With improvements in the effectiveness of systemic treatment and advancements in surgical techniques, there has been a shift to limb reconstruction surgery. For both osteosarcoma and Ewing sarcoma, the current standard of care is multimodal, combining neoadjuvant chemotherapy, surgical resection whenever feasible, and adjuvant chemotherapy, with radiotherapy added for Ewing sarcoma when margins are inadequate, primary tumors are unresectable, or functional considerations favor non-surgical local control [14].

## 2. Materials and Methods

### Literature Search and Selection

**Figure 1:**
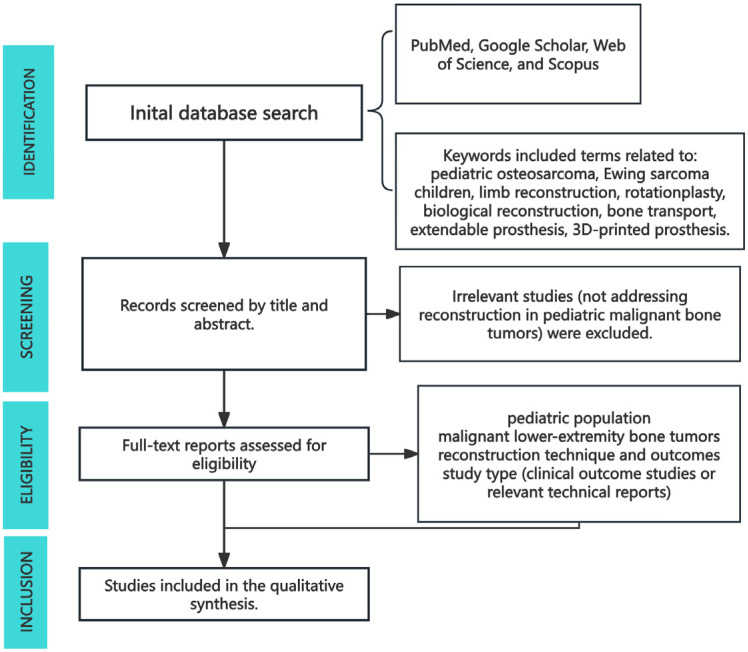
Summary of the search and screening processes used in this narrative review.

This narrative review synthesized published studies on limb salvage and reconstruction strategies for pediatric lower-extremity malignant bone tumors. A comprehensive search was conducted using electronic databases, including PubMed (U.S. National Library of Medicine, National Institutes of Health; Bethesda, MD, USA), Google Scholar (Google LLC; Mountain View, CA, USA), Web of Science (Clarivate; Philadelphia, PA, USA), and Scopus (Elsevier B.V.; Amsterdam, The Netherlands). Keywords and search terms included ‘pediatric osteosarcoma’, ‘Ewing sarcoma children’, ‘limb reconstruction lower limbs’, ‘rotationplasty pediatric’, ‘biological reconstruction children’, ‘bone transport distraction osteogenesis’, ‘endoprosthetic reconstruction pediatric’, ‘extendable prosthesis children’, ‘3D-printed prosthesis pediatric’, and combinations thereof. The search was limited to articles published in English from January 1990 to October 2025 to capture both historical context and recent advances. References in selected articles were manually screened to reduce the risk of missing influential series. We prioritized clinical outcome studies (prospective or retrospective cohorts, case series, and clinical trials) involving skeletally immature patients, but also included selected technical or proof-of-concept reports (e.g., novel extendable or 3D-printed implants) when they described pediatric applications or directly informed growth-preserving reconstruction strategies. Purely preclinical biomechanical or animal studies without clinical correlation were excluded. Because this work was conceived as a narrative synthesis rather than a systematic review, detailed counts of records per database and at each screening stage were not prospectively captured and no formal risk-of-bias assessment tool was applied; outcomes were synthesized descriptively, and the limitations of this approach are acknowledged in the Discussion. An overview of the search and screening process is provided in Figure 1.

## 3. Results

### 3.1. Surgical Options

The surgical treatment of malignant tumors is divided into two distinct parts: tumor resection and reconstruction of the resected site. The basic principle of malignant tumor resection is wide resection of the lesion. After wide resection, the local control rate of the tumor can reach more than 90%. The surgical margins of the bones and surrounding soft tissues are determined on the basis of the preoperative imaging results, taking into account the tumor size, specific anatomical factors, and soft tissue involvement. While a safe tumor-free surgical margin is obtained, the loss of normal tissue is minimized to preserve the best function of the limb [15]. The selection of limb reconstruction surgery for children with lower limb malignant tumors is a complex and highly personalized process that requires comprehensive consideration of the child’s age, tumor location, tumor type, tumor size, chemotherapy effect, and overall prognosis. Limb reconstruction mainly includes rotationplasty, biological reconstruction (such as autologous bone/allogeneic bone transplantation and devitalized bone composite reconstruction), bone transport technology (distraction osteogenesis), and artificial prosthesis reconstruction [15,16].

### 3.2. Rotationplasty

**Figure 2:**
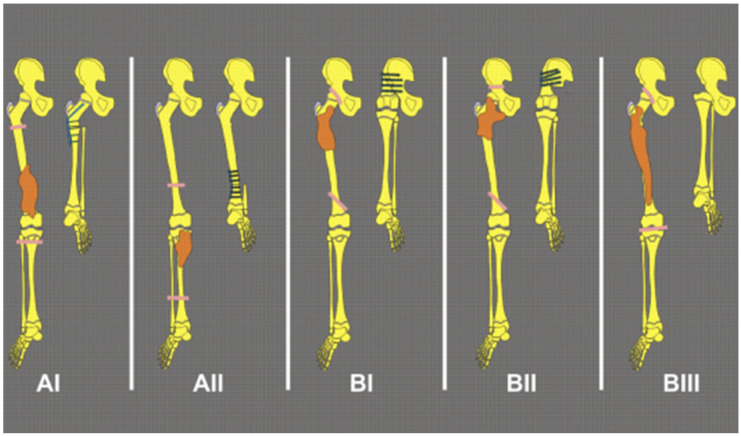
Classification of Winkelmann rotationplasty for lower extremity malignant tumors. (The letters (AI, AII, BI, BII, BIII) denote different classification reconstruction pattern subtype. Orange-colored regions indicate the affected bone segments, whereas yellow-colored regions indicate preserved native bone.).

Rotationplasty was first proposed by Salzer in 1974 as an alternative to above-knee amputation after distal femoral osteosarcoma resection [17]. The core steps of the operation include removing the bone segment affected by the tumor (such as the distal femur or proximal tibia), rotating the distal limb (including the foot) 180° so that the foot faces the back, the ankle joint acts as a new “knee joint”, and reconstructing the limb force line through internal fixation (such as an intramedullary nail or compression plate) [18]. After the operation, the patient can walk with a prosthesis and retain limb sensation and partial motor function [19,20,21]. Winkelmann described a classification of rotationplasty for the treatment of malignant tumors of the lower extremities [22]. Type AI is used for tumors of the distal femur, and type A II is used for tumors of the proximal tibia. Type BI is used for tumors of the proximal femur, type BII is used for tumors of the proximal femur that also involve the hip joint, and type BIII is used for malignant tumors that require resection of the entire femur (Figure 2).

This surgery is commonly used for children with significant growth potential, especially when the tumor is close to the knee joint, and other limb reconstruction surgeries (such as prosthesis implantation) are not feasible [23,24]. Maria Grazia et al. reported that children < 6 years of age (young children) were in a period of rapid bone growth and that the distal femur and proximal tibia contribute significantly to leg length [19]. In addition, children under 6 years of age have greater neurological and psychological plasticity and are more likely to adapt to and accept the biomechanical changes caused by limb rotation. They can learn to use the ankle joint as a knee joint through physical therapy so that they can integrate this change into their sports skills more smoothly than older children can. Therefore, children < 6 years of age are more suitable for rotationplasty [19,25]. The advantages of rotationplasty included a functional “knee”, stable reconstruction, less energy expenditure, fewer future surgeries, and relatively low costs to the patient, making it more affordable [26,27]. Patients who undergo rotationplasty have fewer restrictions in their daily activities due to pain and a much lower complication rate than those who receive an endoprosthesis [28,29]. The main disadvantage is that the reconstruction may be aesthetically pleasing, especially for adolescents and women who have unacceptable cosmetic abnormalities in their anatomy [30]. These negative effects may be overshadowed by the functional improvements achieved with rotationplasty and require careful discussion with the family and child to ensure the right decision and support. Most long-term rotationplasty series include mixed pediatric and young adult populations, with only a subset of patients operated on before the age of 6 years. Cohorts treated for malignant bone tumors typically comprise between 10 and 25 patients (total *n* ≈ 40–60 across the main series), with mean age at surgery ranging from late childhood to young adulthood and mean follow-up of 10–14 years. Reported MSTS scores usually fall between 21 and 28 points (out of 30), and reoperation rates are roughly 20–40%, most frequently for soft-tissue refinement or stump revision rather than failure of the rotationplasty construct [17,23,24,25,26,27,28,29]. Psychological and quality-of-life assessments in long-term survivors suggest that, despite initial cosmetic concerns, many patients—particularly those operated on at a younger age—demonstrate good body image acceptance and post-traumatic growth [19]. Our recommendation to prioritize rotationplasty for children under 6 years thus reflected both this age-related adaptability and the desire to avoid multiple prosthetic revisions during rapid growth, rather than a strict evidence-graded age cut-off.

### 3.3. Biological Reconstruction

#### 3.3.1. Allogeneic/Autologous Transplant Reconstruction

Biological reconstruction refers to the use of autologous or allogeneic bone to repair defects. Common methods include vascularized fibula transplantation, non-vascularized fibula, allogeneic whole bone transplantation, and in vitro devitalized tumor bone transplantation [31].

#### 3.3.2. Vascularized Fibula Graft (VFG)

VFG is mainly used for the reconstruction of intermediate defects (6–15 cm) after the resection of malignant tumors in the lower limbs [32,33]. Karem M et al. reported that VFG was usually the first choice for defects of exactly 6 cm. Even in the case of damage to the tissue bed caused by chemotherapy or radiotherapy, its blood supply can ensure rapid healing and initial healing [34]. Sabina M et al. reported a cited healing rate of 82.4% (approximately 14 of 17 reconstructions) and the mean time to union of 10.6 weeks were derived from a mixed adult–pediatric cohort rather than from children specifically, and most nonunions occurred in adults with larger defects or prior radiotherapy [35]. Although the study included patients as young as 3 years old, the authors did not provide a discrete pediatric subgroup analysis, and the exact number of pediatric patients contributing to these outcomes was not reported. When combined with allografts or frozen autografts, VFG can provide both biological activity (vascularized bone) and mechanical strength, reducing the risk of nonunion and fracture [36]. Its advantages are high biological activity and adaptability to complex anatomical requirements, but the indications (such as defect length, location, and soft tissue conditions) must be strictly controlled. It can be combined with other techniques (such as external fixation and flaps) to reduce the risk of complications [34,37].

#### 3.3.3. Non-Vascularized Fibula Graft

Non-vascularized fibula grafting (NVFG) is a biological reconstruction method used to repair large bone defects caused by the resection of malignant bone tumors in the lower limbs. Compared with vascularized grafting, NVFG technology is simple to perform, does not require microsurgery, has a short intraoperative time, and has fewer donor site complications. It is suitable for children with small bone defects (generally <6 cm). Sheridan et al. reported 10 cases of pediatric malignant bone tumors that were reconstructed via the NVFG. The average follow-up was 56 months, with a bone healing rate of 100%, no graft failure, and good functional scores [38]. Moreover, in Lenze et al.’s series, 12 pediatric lower-limb cases (age 5.5–17.7 years) underwent NVFG reconstruction of femoral, tibial, or fibular defects measuring 6.5–24 cm. Most achieved radiographic union within 7–47 weeks, whereas one delayed union (61 weeks) and one non-union occurred exclusively in large segmental defects ≥ 12 cm combined with chemotherapy. Across all lower-limb junctions in this subgroup, primary union was consistently high, reflecting the overall study’s 94% union rate. Defect length—not age—was the primary determinant of delayed or failed consolidation [39].

#### 3.3.4. Allogeneic Whole Bone Transplantation

Allografts are a biological method commonly used for the reconstruction of large bone defects. It uses a whole piece of frozen or freeze-dried bone from a donor to restore bone structure and mechanical integrity. Its advantages are that it can reconstruct longer defects (>6 cm), does not require the collection of autologous bone, and has few donor site complications; however, the risks include graft absorption, nonunion, and immune-related complications [40]. It is used in the reconstruction of lower limb malignant tumors (such as those of the distal femur and proximal tibia).

#### 3.3.5. Ex Vivo Inactivation of the Tumor Bone

Extracorporeal devitalized bone replantation is a method of removing the tumor bone segment, devitalizing it in vitro (such as by radiotherapy, liquid nitrogen freezing, pasteurization, etc.), and then replanting it to its original location. This technique aims to preserve the patient’s own bone tissue, reduce immune rejection, and provide a feasible reconstruction solution in areas with limited economic value. It is particularly suitable for reconstruction after interstitial resection [41,42,43]. The bone healing rate after liquid nitrogen devitalized autologous bone replantation can reach more than 80%, and the average healing time is 6–12 months [41]. The local recurrence rate after devitalized bone replantation is 8.7–10%, which is more advantageous than artificial prosthesis reconstruction (15–30%) [44]. Long-term follow-up (average of 8–14 years) revealed that the graft survival rate was stable [42]. The infection rate is approximately 10.8%, and the incidence of bone nonunion is approximately 21% (mostly around the knee joint). The risk can be reduced by the use of antibiotic bone cement or combined vascularized transplantation [45].

#### 3.3.6. “Hot Dog Technique” Composite Reconstruction

The so-called “hot dog technique” or Capanna technique uses a large section of allogeneic bone or autologous devitalized bone as the outer layer and inserts a vascularized autologous fibula as the inner core, providing sufficient mechanical support and blood supply growth potential through the composite of the dual structure. This autologous/allogeneic hybrid construction can promote the ossification and integration of the bone graft while ensuring structural strength. When the defect length exceeds 15 cm (large defects), the bone quality is poor or the revision fails, the “hot dog” technique can be preferred [46,47]. Findings from a recent systematic review and meta-analysis (2025) further refine the clinical implications of this approach. The study demonstrated that Capanna reconstruction achieves overall complication rates and functional outcomes comparable to VFG alone; however, in adults, the Capanna construct significantly reduces fibular graft fracture rates (17.2% vs. 35.0%, *p* = 0.03), underscoring its mechanical superiority in high-load environments. Notably, this benefit comes at the cost of a higher reoperation rate (49.6% vs. 26.5%, *p* = 0.005), reflecting the inherent procedural complexity and the potential need for secondary interventions such as additional grafting or hardware revision. Importantly, the same meta-analysis identified adjuvant chemotherapy as a strong independent predictor of nonunion (OR 10.1, *p* < 0.0001), regardless of reconstructive method, highlighting the value of constructs capable of providing both robust mechanical support and enhanced biological healing capacity in patients undergoing systemic therapy [48]. Taken together, recent evidence reinforces the role of the hot dog technique as a preferred reconstructive strategy for large segmental defects—particularly in the lower extremity—where high mechanical demands and chemotherapy-related impairment of host healing capacity necessitate a reconstructive approach that integrates structural stability with vascularized biological support [48,49].

### 3.4. Bone Transport Technology

Bone transport (distraction osteogenesis) technology uses an external fixator to gradually distract the osteotomy site and gradually fill the bone defect [50]. It is suitable for large bone defects (usually 3–15 cm) after the resection of malignant tumors in the lower limbs of children, especially when the epiphysis can be preserved, to reconstruct bone defects and correct limb length discrepancy (LLD) [51,52]. Tumor-related bone transport series using the Ilizarov method typically include adolescents and young adults, with pediatric patients comprising roughly one-third to one-half of the sample (age range approximately 8–25 years) [50,51,52,53,54]. Bone transport technology generally requires long-term external fixation (usually several months) and multiple surgeries (such as external fixator adjustment and treatment of complications such as screw tract infection) [31,53]. Union is achieved in nearly all cases, but at the cost of prolonged treatment: external fixation indices often exceed 40–60 days/cm, and patients undergo on average 1–2 unplanned additional procedures. Pin-tract infections occur in about 50–60% of patients, joint stiffness in roughly 20–30%, and regenerate or docking-site problems requiring intervention in 10–20% [51,52,53]. These burdens are particularly challenging in younger children, in whom compliance with long-term external fixation can be limited. A study reported that the average external fixation time for patients who used the Ilizarov method to reconstruct osteosarcoma resection defects was more than 34 months, and approximately 1.7 additional surgeries were required to address complications, with a limb preservation rate of approximately 90% [53]. Chemotherapy also has a certain effect on bone healing after bone transport. Anthony et al. [54] reported a prospective study and reported that all patients (including children) who underwent bone transport surgery achieved bone healing and full weight bearing. All 30 patients achieved bone healing within 1 year after surgery, with a success rate of 100%. The healing speed of bone segments that did not receive chemotherapy was significantly faster, and the healing time of the chemotherapy group was twice that of the nonchemotherapy group. The effectiveness and safety of bone transport vary with age. For children under 10 years of age, bone transport technology is more challenging because their epiphyses have not closed. Although bone transport can preserve limbs, it has a long treatment cycle and many complications (infection, nonunion, etc.) and requires high compliance from children, so it is often used as a last resort.

### 3.5. Endoprosthesis Reconstruction

In limb-sparing reconstruction for treating malignant tumors of the lower limbs in children, prosthetic reconstruction has become one of the most important methods for repairing mechanical bone defects. The most common site for prosthetic reconstruction is the distal femur, accounting for approximately 70–75%, followed by the proximal tibia, accounting for approximately 15–20%, and a few are the proximal femur. Recent series of non-invasive growing prostheses in children report a 5-year revision-free implant survival of approximately 61.6–100% [55,56], while large cohorts of predominantly invasive or mixed expandable systems demonstrate comparable 5-year prosthesis survival rates in the range of 59.4% [57]. Yuan, Li et al. reported 49 children with proximal tibial osteosarcoma who underwent knee prosthesis replacement, with a 5-year survival rate of 83.2% (41/49) and a total prosthesis survival rate of 87.4% (43/49) after 5 years [58]. The main prosthesis replacement options include standard modular prostheses, extendable prostheses, semi-joint/full-joint customized prostheses, bone-prosthesis composite reconstruction, and 3D-printed prosthesis reconstruction.

#### 3.5.1. Modular Prosthetic Reconstruction

A modular limb prosthesis consists of a set of prefabricated, interchangeable components—typically a diaphyseal stem, cylindrical segmental spacers of various lengths, and an articulating joint module—that can be assembled intraoperatively to match the resection length and joint level. After cemented or press-fit fixation of the stem, the surgeon stacks segmental spacers and attaches the knee or hip component to restore limb length and immediate mechanical stability, allowing early weight-bearing. Modular prostheses can achieve almost all reconstructions from the proximal femur to the distal femur and are suitable for reconstruction after epiphyseal closure or near-closure of the epiphysis and for small-expected bone tumor resection [15]. The main advantages of modular prosthetic implants include convenient postoperative rehabilitation, joint stability, immediate weight-bearing, and rapid limb function recovery. However, prosthetic complications remain a major concern in growing patients. Infection, aseptic loosening, mechanical failure, and periprosthetic bone resorption are consistently reported as leading causes of implant failure [32]. A study by Giovanni et al. revealed that the survival rate of pediatric patients treated with 3D-printed prostheses 2–5 years after surgery can reach more than 85%, and the main complications include periprosthetic infection (approximately 9%) and mechanical loosening (approximately 7%) [58]. The survival rate of the prosthesis (freedom from any revision vs. freedom from aseptic loosening) gradually decreases over time and is approximately 50% to 80% within 5 to 10 years, which is related to the length of time after surgery, the reconstruction site, and the amount of bone resection [59,60,61].

#### 3.5.2. Extendable Prosthesis

Extendable prostheses offer a key solution for limb length discrepancy (LLD) in skeletally immature patients. These prostheses compensate for the natural growth of the contralateral limb via noninvasive postoperative lengthening (e.g., electromagnetic or mechanical devices) and are particularly useful for reconstruction after distal femoral or proximal tibia tumor resection [62,63]. A feature of these devices is that the endoprosthesis can be extended like a telescope. The length that can be achieved is proportional to the amount of bone removed; the longer the prosthesis is, the greater the lengthening capacity. These extendable components have become popular and offer an alternative to amputation and rotationplasty in growing children. However, prostheses are associated with a high rate of wear and failure [64,65]. Many studies have shown that patients who receive extendable prostheses require multiple surgeries. Noninvasive extendable endoprostheses typically allow lengthening by approximately 3–5 mm per session, performed every 3–6 months according to growth velocity, with total planned lengthening often in the range of 2–5 cm over childhood. The number of lengthening surgeries ranged from 6.4 to 8.4 per child, with 5 of 6 and 25 of 54 patients undergoing multiple surgeries for limb lengthening and prosthesis revision surgery, replacement of prosthesis failure, replacement with a larger prosthesis, aseptic loosening of the prosthesis, and infection, respectively [62,63,64,65,66,67]. Overall, between half and two-thirds of patients required at least one unplanned reoperation beyond scheduled lengthening.

#### 3.5.3. D Printing Synthesis

The application of 3D-printed prostheses in limb reconstruction of malignant tumors in children with lower limbs is an innovative treatment method that aims to provide children with personalized bone substitutes. It is mainly used in special situations, such as complex bone defect morphology, difficult matching of conventional modular prostheses, the need to retain joints or growth plates, and failed repair and reconstruction [68,69,70,71]. 3D printing technology allows the design and manufacture of highly fit prostheses because of the specific anatomical structure and tumor resection range of the child, thereby improving the accuracy of surgery and functional recovery. Zhao, Zhang et al. reported a retrospective study of 28 patients, of which 14 (12–75 years old) used 3D-printed prostheses and 14 (10–54 years old) used allogeneic bone transplants. Of the 28 patients, 8 were under 18 years [72]. The incidence of postoperative complications such as bone nonunion, graft fracture, and infection was as high as 50% in the allogeneic bone group, whereas it was only 7% in the 3D-printed prosthesis group. The average bone healing time in the 3D-printed prosthesis group was 6.1 months, which was much faster than the 12.2 months reported in the allogeneic bone transplant group [72]. Children under 6 years of age have strong potential for epiphyseal growth. To preserve the function of the growth plate as much as possible, 3D-printed prostheses should be selected with caution. For children aged 6–15 years, joint-preserving 3D-printed prostheses can be selected. Taojun Gong et al. reported 7 children with an average age of 11 years who underwent joint-preserving surgery and applied modular 3D-printed prostheses. The average follow-up was 30 months (27–59 months). The average Musculoskeletal Tumor Society (MSTS) score after surgery was 17 points, which improved to 28 points after surgery. Complications such as prosthesis migration and fracture or periprosthetic fracture were not observed [68]. Its advantage is that the articular surface and epiphysis are preserved, and the growth potential is maintained to the greatest extent.

### 3.6. Specific Body Part Problems

The most common site of osteosarcoma in children is between the mid-femur and mid-tibia. However, the treatment plan and results for other parts of the lower limb may differ. The pelvis, ankle joint and hindfoot regions have greater requirements for limb-sparing treatment because of their complex anatomical structure, special weight-bearing ability and activities. In recent years, with the rapid development of imaging, navigation, minimally invasive and other technologies, significant progress has been made in limb-sparing surgery in these special parts. The application of computer-assisted navigation in the resection of malignant tumors in the lower limbs of children is gradually increasing. Based on the patient’s CT data, a three-dimensional model is constructed, and the real-time positioning of the navigation device is combined during the operation to ensure that the tumor resection margin is consistent with the plan and to maximize the preservation of important bone structures. Hwan Seong et al. reported 3 patients whose tumor margins were accurately determined through computer-assisted navigation. Tumor resection can achieve an error of less than 1 mm, and the postoperative recurrence rate and functional score are better than those of traditional open surgery [73]. Weiyi, Wang et al. reported a case of an 11-year-old patient with a malignant tumor of the talus who underwent limb reconstruction with a 3D-printed prosthesis [69]. At the 12-month postoperative follow-up, the patient had good functional recovery, a Musculoskeletal Tumor Society (MSTS) score of 27/30, an American Orthopedic Foot and Ankle Society (AOFAS) score of 92/100, a good prosthesis position, and no narrowing of the joint space [69]. In growing children, adjusting the size of the femoral head allograft to match the acetabulum is challenging. Hemiarthroplasty is usually used for the reconstruction of pediatric proximal femoral tumors. Manoso et al. described the changes in the acetabulum after hemiarthroplasty [74]. In the absence of a growing femoral head, the normal deepening and widening of the acetabulum ceased. These patients experienced progressive superolateral displacement of the prosthetic femoral head over time, which was more obvious in younger patients. The feasibility of limb-salvage surgery in specific areas depends on strict case selection criteria to ensure tumor control and functional recovery. Patients who are sensitive to radiotherapy and chemotherapy, whose tumors have significantly shrunk, and whose tumors have not invaded major blood vessels or nerves or caused pathological fractures are more suitable for limb salvage surgery. For malignant tumors in the foot and ankle, adequate soft tissue coverage should also be considered, which is crucial for wound healing [75].

### 3.7. Long-Term Growth Monitoring

All patients require annual limb-length scans (teleoroentgenogram or EOS) until skeletal maturity is reached. Growth prediction should be based on established methods, such as the Paley multiplier method and Menelaus ‘rule of thumb’, which estimate remaining growth at each physis. For example, complete arrest of the distal femoral physis in a 6-year-old boy is expected to produce an untreated limb-length discrepancy of roughly 8–9 cm at maturity, given that this physis contributes approximately 9 mm of growth per year until closure. In our algorithm, a predicted limb-length discrepancy > 4 cm at maturity warrants contralateral epiphysiodesis and/or staged lengthening, with the exact threshold individualized according to functional demands and comorbidities. Psychological support is essential throughout growth, particularly for rotationplasty patients during adolescence.

Decision-Making Algorithm for Pediatric Patients

**Figure 3:**
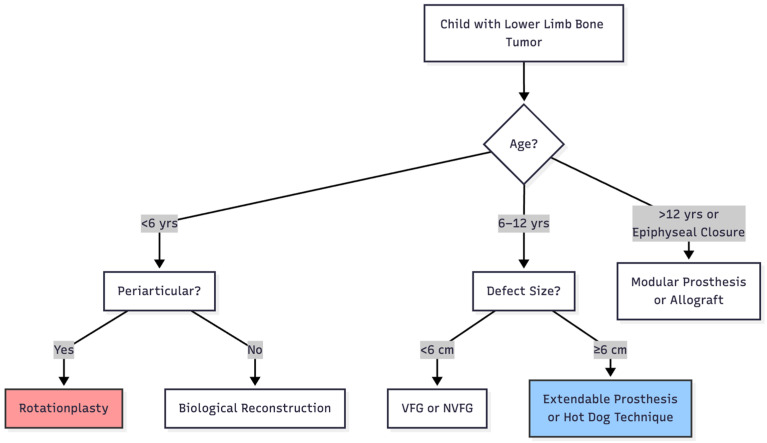
Proposed treatment algorithm for limb reconstruction in pediatric lower extremity malignant bone tumors.

A practical treatment algorithm is proposed (Figure 3):

## 4. Discussion

This narrative review synthesized contemporary limb-salvage strategies for pediatric lower-extremity malignant bone tumors, prioritizing comparative interpretation rather than reiteration of numerical outcomes. In skeletally immature patients, reconstructive decision-making is governed predominantly by three variables: patient age, defect length, and the feasibility of physeal preservation. In addition, we acknowledge that the majority of the available evidence is derived from retrospective, single-center cohorts with inherently limited sample sizes. We did not apply a formal risk-of-bias tool or perform a quantitative meta-analysis; instead, we synthesized outcomes descriptively to reflect the heterogeneity and variable quality of the existing literature. Accordingly, the proposed algorithm should be interpreted as an expert-informed conceptual framework rather than a formally graded, evidence-based guideline.

Rotationplasty offered reliable long-term function, low complication rates, and minimal need for revision in children under 6 years of age. Its biomechanical efficiency and superior MSTS scores supported its use as a first-line option when joint-preserving biological reconstruction or extendable prostheses are not feasible. Although cosmetic concerns may arise during adolescence, rotationplasty maintained excellent stability and energy efficiency, helping many patients return to high-demand activities.

Biological reconstruction techniques—including non-vascularized fibula grafts (NVFG), vascularized fibula grafts (VFG), allografts, and the Capanna “hot dog” composite—were most appropriate for children with substantial remaining growth. NVFG was typically used for defects < 6 cm and demonstrated high union reliability. VFG and combined constructs were preferred for 6–15 cm defects or for metaphyseal reconstructions in which biological remodeling is desirable. Although biological methods require longer healing (6–12 months), they avoided the mechanical complications frequently observed in endoprosthetic reconstruction and preserved long-term growth potential.

Bone transport addressed large defects (3–15 cm) when joint preservation is possible. It reliably achieved union but carried a high burden of prolonged external fixation, pin-tract infection, and multiple unplanned surgeries. Thus, it was best reserved for selected patients, including those in whom prosthetic options are limited or contraindicated.

Endoprosthetic reconstruction—including modular, extendable, and 3D-printed prostheses—was most suitable for children aged 6–12 years (School-aged and early adolescents) who require periarticular resection with loss of the physis. Extendable prostheses were effective for controlling limb length discrepancy but often required multiple lengthening procedures and revisions. Emerging 3D-printed joint-preserving implants showed favorable early outcomes, reduced complication rates, and improved epiphyseal protection, especially in irregular defects not suitable for standard modular implants.

In practical surgical pathways, age is one of the primary determinants of reconstruction choice. As such, management can be generally categorized as follows:

Young children (<6 years): rotationplasty and biological reconstruction strongly considered, especially for periarticular tumors near the knee.

School-aged and early adolescents (6–12 years): mixed strategies; biological reconstruction for small/intermediate defects and extendable or joint-preserving 3D-printed prostheses for larger resections.

Older adolescents (>12 years): modular (non-extendable) prostheses and joint-sacrificing reconstructions with more stable long-term mechanics.

To improve clarity and avoid redundancy, Table 1 consolidates key indications, defect ranges, functional outcomes, and complication profiles across major reconstructive strategies. The Discussion therefore focuses on interpreting these differences and explaining how they inform clinical decision-making. Age remains the central variable: younger children benefit most from biological reconstruction or rotationplasty, whereas older adolescents whom near-closure of growth plates are better suited to modular or extendable prostheses. Predicting final LLD and planning for contralateral epiphysiodesis (>4 cm predicted discrepancy) are critical components of long-term management.

Future directions include biomimetic scaffolds, vascularized composites, and magnetic lengthening systems aimed at reducing revision rates and improving biological integration. As most available studies are retrospective and heterogeneous, further prospective pediatric-specific research is needed to strengthen evidence-based reconstruction algorithms.

## 5. Conclusions

The diagnosis and treatment of childhood osteosarcoma face many challenges, especially in limb salvage and reconstruction. The complexity of surgical options and growth characteristics require doctors, patients and their families to participate in full discussion. Preoperatively, the reconstruction plan should be formulated by comprehensively considering factors such as tumor location, defect length, child age and expected growth. The reconstruction method used is determined on the basis of the length, location and age of the resection. When the bone defect is small, biological reconstruction is preferred, which can be used for long-term survival; when the defect is large, extendable prosthesis should be considered; if the age is too young for proximal femoral and proximal tibia tumors, rotationplasty can be preferred; if the risk of limb salvage is too high, amputation should be considered. In the future, new technologies such as personalized 3D-printed prostheses or extendable prostheses may be used to further improve limb reconstruction strategies for children. In addition, the personalized needs of children and their families should be fully considered. Ultimately, choosing a personalized treatment plan will help improve patients’ quality of life and survival expectancy.

## Figures and Tables

**Table 1:** Overview of main reconstructive strategies for pediatric lower-extremity malignant bone tumors.

Technique	Typical Age Band	Defect Range	Main Indications	Typical Union/Prosthesis Survival	Key Complications
Rotationplasty	Mainly <6 years (also older children/adolescents)	Periarticular resections	Distal femur/proximal tibia tumors when joint-sparing or extendable prosthesis is not feasible	MSTS usually 21–28/30; good long-term function and return to sports	Cosmetic concerns; occasional stump revision
NVFG/small allograft	Children of all ages	<6 cm	Small diaphyseal defects	Union > 80%; mean union ≈ 4–8 months	Graft fracture; donor-site morbidity
VFG ± “hot dog”	Mostly 6–15 years	6–15 cm (selected > 15 cm)	Larger diaphyseal/metaphyseal defects with growth potential	Union ≈ 82–95%; mean union ≈ 6–12 months	Stress fractures; ankle morbidity; nonunion (15–30%)
Bone transport	Broad (school-age to adolescents)	3–15 cm	Large defects with preserved joints	Union in nearly all patients; external fixation index ≈ 40–60 days/cm	High pin-tract infection (50–60%), joint stiffness, regenerate problems
Modular/extendable prosthesis	6–12 years (extendable); adolescents (fixed modular)	Usually >6–8 cm	Periarticular resections removing physis	5-year prosthesis survival typically 54–87% (definitions vary); MSTS ≈ 70–85%	Infection (10–15%), aseptic loosening (15–28%), mechanical failure
3D-printed joint-preserving	~6–15 years	Variable, often metaphyseal	Irregular defects, need for epiphyseal preservation	Early series: union in most cases; mean union ≈ 6.1 months vs. 12.2 months for allografts; MSTS 17→28/30 in 7-child series	Limited follow-up; rare early mechanical complications

NVFG: Non-vascularized fibula grafting. VFG: Vascularized Fibula Graft.

## Data Availability

No datasets were generated or analysed during the current study.

## Abbreviations

The following abbreviations are used in this manuscript:

AOFAS	American Orthopedic Foot and Ankle Society
EWS	Ewing sarcoma
LLD	Limb length discrepancy
MSTS	Musculoskeletal Tumor Society
NVFG	Non-vascularized fibula grafting
OSC	Osteosarcoma
SEER	Surveillance, Epidemiology, and End Results
VFG	Vascularized Fibula Graft
